# “I Heard of PrEP—I Didn’t Think I Needed it.” Understanding the Formation of HIV Risk Perception Among People Who Inject Drugs

**DOI:** 10.1007/s11013-024-09870-8

**Published:** 2024-07-15

**Authors:** Sarah Mars, Jeff Ondocsin, Kimberly A. Koester, Valerie Mars, Gerald Mars, Daniel Ciccarone

**Affiliations:** 1 Department of Family and Community Medicine, University of California, San Francisco, 490 Illinois Street, San Francisco, CA 94158 USA; 2 Center for AIDS Prevention Studies, University of California, San Francisco, 550 16th St, San Francisco, CA 94158 USA; 3 Department of Anthropology (Honorary), University College London, Gower St, London, WC1E 6BT UK

**Keywords:** Injecting drug use, HIV, Risk perception, Pre-exposure prophylaxis, Cultural theory, Modes of social organization

## Abstract

Uptake of pre-exposure prophylaxis medication (PrEP) to prevent HIV among people who inject drugs (PWID) remains extremely low in the United States. West Virginia’s rising HIV incidence and highest drug overdose rate in the nation makes it an important locus for opioid use and HIV risk interaction. In this pilot study we pioneered the use of Cultural Theory among PWID to understand HIV-related risk perception arising from four contrasting modes of social organization. Carried out during an HIV outbreak, we explored PrEP uptake qualitatively as a window onto risk perception. Of the 26 interviewees, 18 were HIV− and despite the medication’s free availability from the health center where recruitment took place, none had taken PrEP, half considering they were not at risk. Intimate couples who showed characteristics of ‘enclaves’ considered the boundary around themselves protective against HIV, creating a safe space or ‘invisible risk group’. Higher HIV risk was perceived among those who were housed compared to those living homeless. Beliefs about the causation of the local HIV outbreak and the validity of scientific research corresponded with characteristics of the contrasting modes of social organization and the approach is promising for informing public health interventions among PWID.

## Introduction

This study uses questions about pre-exposure prophylaxis (PrEP), medication that can prevent HIV transmission, as a window onto risk perception among people who inject drugs (PWID). We explore how and why people injecting drugs form their assessments of their risk of acquiring HIV.

With the US opioid epidemic’s spread and rising numbers of people injecting drugs, HIV transmission through contaminated injecting equipment has risen by 12% nationally (2017–2019) with many local outbreaks (AHEAD, 2022; Peters et al., 2016; Evans et al., 2018; Centers for Disease Control & Prevention, 2018). HIV disproportionately affects people of color with prevalence rates more than five times higher among black than white men and more than 14 times higher among black women than white (Emory University Rollins School of Public Health GS, Inc. and the Center for AIDS Research at Emory University (CFAR), 2022). Recent HIV outbreaks in West Virginia add to the existing risk of overdose among people using drugs, the highest rate in the nation at 81.4 per 100,000 standard population in 2020 (Hedegaard & Warner, 2020). From 2017 to 2019, HIV incidence increased by 90% in the state (AHEAD) AsHEAD, 2022). The downward national trend in new cases of HIV in the US is welcome news but does not apply to all transmission routes.

Located in the Appalachian region of the United States (US), WV is a largely rural state with significant differences between localities in measures of social cohesion, community trust and interpersonal relationships (Bell, 2009). Coyne et al. showed the dynamic nature of southern WV culture with the endurance of traditional values such as religious belief and strong family ties alongside changing gender relations and decision-making processes (Coyne et al., 2006).

PrEP has proven effective in preventing HIV infection through sexual transmission while evidence for its prevention of parenteral transmission is encouraging but less strong (Choopanya et al., 2013; Grant et al., 2010). Among risk groups accessing PrEP, PWID are under-represented and there may be particular challenges in reaching and engaging this population (Coleman & McLean, 2016; Garner et al., 2018). In the general population, PrEP uptake, although increasing, varies greatly by region and gender. In 2019, women made up only 7.4% of PrEP users but 19% of newly diagnosed HIV cases. New York state leads uptake with 187 people per 100,000 and Wyoming last with 22 per 100,000 (Emory University Rollins School of Public Health GS, Inc. and the Center for AIDS Research at Emory University (CFAR), 2022). In one study, young gay and bisexual men using PrEP had travelled along a continuum of knowledge acquisition prior to initiation, corresponding to existing values and information sources (Koester et al., 2021).

A recent systematic review noted that while PrEP awareness and willingness was higher than previously thought among PWID, uptake was still very low (0–3%) (Mistler et al., 2021). Some studies have found low levels of knowledge about PrEP among PWID but also high levels of willingness to use it once informed (Sherman et al., 2019; Walters et al., 2017), particularly among women (Patel et al., 2019; Roth et al., 2018) and people with greater educational attainment (Egorova et al., 2021).

Rohrmann and Renn define risk perception as “people’s judgments and evaluations of hazards they (or their facilities, or the environment) are or might be exposed to” including both experiences and beliefs (Rohrmann et al., 2000). The significance of HIV risk perception in PrEP uptake, while acknowledged, remains poorly understood. Among people who use drugs, the perception of being at any risk for acquiring HIV has been associated with greater willingness to use PrEP but the process of forming risk perception is unclear (Stein et al., 2014).

Clinical models for pre-exposure prophylaxis refer to the PrEP ‘cascade of care’, a series of steps for identifying, engaging and retaining individuals in treatment, starting with “(1) identifying individuals at highest risk for contracting HIV, (2) increasing HIV risk awareness among those individuals and (3) enhancing PrEP awareness” (Nunn et al., 2017). Most research on what is seen as a progressive continuum uses a positivist framework. Accordingly risk is considered an objectively measurable probability, an inaccurate perception of which results from a knowledge deficit; recommendations for further education then follow this model e.g. Ergorova et al. (2021).

Qualitative research among US PWID has found multiple barriers to PrEP uptake including individual-level issues such as low PrEP knowledge, concern about side-effects and the competing priorities of daily drug use; inter-personal level barriers such as experiences of HIV related stigma among social networks and health care providers and structural barriers such as the practical difficulties of homelessness and criminal justice system involvement disrupting medication adherence (Biello et al., 2018). Research among homeless youth injecting drugs in Canada found ambivalence towards PrEP use based on low risk perception, concerns about practical difficulties for adherence and misonceptions about HIV transmission based on personal hygiene (Dahlby et al., 2022). Other researchers in the US Northeast, noting the wide range of risk perception of HIV transmission among PWID, conclude that, “Understanding the reasons for low perceived HIV risk in specific PWID populations, which could relate to younger age, low knowledge of HIV transmission, denial, or other factors will be important for developing PrEP uptake interventions.” (Bazzi et al., 2018).

### Theoretical Framework

Variations in individuals’ assessment of whether particular hazards are to be tolerated, managed or avoided have been explained by multiple hypotheses, including technical knowledge about risks, cultural variations, political orientation and personality. The study of other cultures undertaken by anthropologists has provided important insights into how uncertainty and risk are approached across time and place (Alaszewski, 2015). They have shown cultural variation to be important in the assessment and conception of risk and in shaping health behavior (Helman, 1978). Behavioral responses to health advice depend on culturally derived beliefs about the source and likelihood of infections, definitions of ‘community’ and ‘outsiders’ as well as the perceived legitimacy and trustworthiness of information sources (Bish & Michie, 2010). It is therefore important to understand these contrasting cultures for the development of effective HIV prevention interventions.

**Fig. 1:**
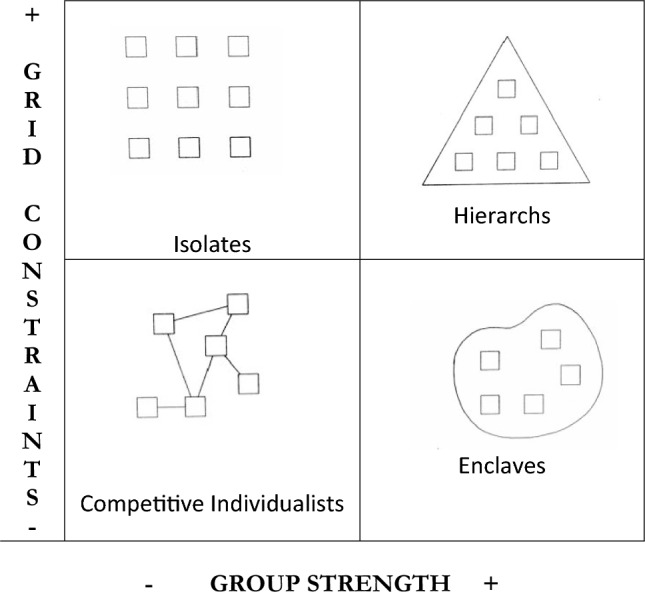
Four modes of social organization. Adapted from Frosdick and Mars, 1997 with permission (Frosdick & Mars, 1999)

The definition of ‘culture’ used here takes it to mean ‘ways of life’ comprising patterns of interpersonal relations, shared values and beliefs (Thompson et al., 1990). Cultural Theory, developed by British anthropologist Mary Douglas and rooted in the work of founding sociologist Emile Durkheim, measures two dimensions of social structure and relations: the level of prescriptiveness of norms or rules (‘grid’ constraints) and the degree of affiliation between individuals, social cohesion and encircling boundaries (‘group’), which, in differing combinations produce four distinctive modes of social organization (see Fig. 1) which in turn explain beliefs and behaviors (Bloor & Bloor, 1982; Douglas, 1978). Although these modes may not exist in this form in the empirical world, they are helpful in guiding the application of the theory. These four contrasting cultures express corresponding attitudes to time, knowledge, the body, sources of authority, and perceptions of risks (Douglas & Wildavsky, 1982). They have been found to co-exist within societies, often in competition with each other and may be any size (Mars, 2021; Adams, 1995). Unlike some ethnographic approaches that are limited by the specificity of the cultural context they describe, using these two universal characteristics allows us to make cross-cultural comparisons. It also produces a more differentiated analysis of differently organized individuals who are frequently otherwise defined from the outside by their ethnicity, material conditions or stigmatized status.

In 1990 Mary Douglas and French sociologist Marcel Calvez published, ‘The Self As Risk Taker. A Cultural Theory of Contagion in Relation to AIDS’ (Douglas & Calvez, 1990). The paper addresses two areas in relation to contagion: (1) the social and political argument involved in the creation of a city community and (2) the social experience in the context of a communicable disease in which Calvez proposed two possible layers of individual protection from HIV: the individual body and the community (Calvez, 2011). The authors proposed contrasting ideas of where HIV transmission risk would be located, exploring different perceptions and responses to this risk. This second part forms the basis of the theoretical approach used in the West Virginia study.

In their largely theoretical paper, Douglas and Calvez propose several contrasting ideas of where HIV transmission risk is located in relation to the body according to the four main modes of social organization. Hierarchical cultures with many rules (strong grid) and a clear perceived boundary (strong group) express trust in professional scientific knowledge and promote risk-avoidance as protection against HIV transmission. In an egalitarian group or ‘enclave’ there are fewer rules but stronger boundaries towards the outside (weak grid, strong group). They tend to be more skeptical of established scientific knowledge and advice, while identifying their own community as protective, keeping out potentially contaminating outsiders and expelling ‘at-risk’ insiders. Strong group cultures view the community as a second ‘skin’ which must be safeguarded against such contamination, its members sometimes prioritizing protection of the community boundary over protection of the body itself.

We piloted the use of Cultural Theory among PWID in the US and consider whether Douglas and Calvez’ predictions correspond with those found decades later in West Virginia. At the time of the research visit, there was a local HIV outbreak and this resulted in particularly intriguing data. Douglas and Calvez assert that individualists who reject membership of groups and adherence to rules (weak grid and weak group) are more likely to be risk takers by expressed preference, considering themselves able to control their own risk successfully. Finally, ‘isolates’ or ‘fatalists’, controlled by the rules of others and isolated from group membership (strong grid, weak group), tend to adopt a fatalistic explanatory framework, reflecting a perceived lack of control over their lives. Earlier findings from this current WV study used this theoretical approach to explain why HIV-related stigma had greatly diminished among PWID since the local outbreak. Fatalism, particularly among single people living homeless, undermined individuals’ aspirations towards mutual support but also contributed to tolerance and acceptance of HIV positivity (Mars et al., 2022).

### Locating Ourselves as Researchers

A valuable aspect of viewing research subjects through the lens of Cultural Theory is that it also enables a much greater degree of reflexivity in the researchers. Rather than simply reflecting on one’s own relative economic or social priviledge, this framework allows us to place ourselves within a particular set of values and behaviors, each with their own strengths and weaknesses, along with concommitant attitudes towards other modes of organization. Douglas classifies scientific expertise and public health approaches in western societies as emanating from individualists and hierarchs (those adhering to a hierarchical social structure) as part of the central establishment. Knowledge is trusted by hierarchs and used by individualists when produced by established organizations staffed by those with accredited status gained from long-term study, such as universities and the professions. As creators of such knowledge within these institutions, we the authors largely adhere to these values. When assessing risks of HIV transmission, we would point to the higher levels of HIV infection among PWID using scientific evidence and from there conclude that PWID are at higher risk, particularly during an outbreak of HIV where the prevalence is elevated.

Greater skepticism of these forms of knowledge would be expected from those who separate themselves from the establishment center by choice because they dislike its norms, forming egalitarian, bounded enclaves or those expelled to the margins by the establishment centre, controlled by others and by structural inequalities as isolated, constrained individuals. These relationships are therefore both social and political. There is tension and movement between those adhering to the center and those alienated from it. Accordingly Douglas describes a ‘negative diagonal’ of fatalists and enclaves that rejects or is rejected by the ‘positive diagonal’ of power connecting individualists and hierarchs. This process is evident in the challenges made to public health and its scientific knowledge base in recent years and the successful encroachment upon it by those rejecting such knowledge (Latkin et al., 2021; Rutjens et al., 2022).

## Methodology

A Syringe Service Program (SSP) in a West Virginia town hosted the research project for study recruitment. The team had some familiarity with West Virginia following an earlier research visit (Ondocsin et al., 2020). Ethnography is the traditional method for observing cultural characteristics in a community. However, research on an individual basis can also be a viable method of capturing cultural attributes. In September 2019 1 week of intensive qualitative research was carried out by three of the authors and additional team members visiting WV. We conducted 26 semi-structured interviews with PWID during this visit (Guest et al., 2006).

### Preparation

After initially piloting and refining the interview guide at Homeless Youth Alliance, a San Francisco SSP, we revised the guide for the field site. Most of the questions, which were aimed particularly at the ‘group’ dimension, worked well in the WV context. We allowed features of ‘grid’ to emerge through thick description. We revised the guide every morning of the research according to experiences on the previous day.

We included in the interview guide conceptions of ‘family’; trust and mutual reliance; whom participants spent time with; individual risk perception and behavior around opioids and blood borne viruses; awareness, preference and uptake of PrEP; sexual behavior including transactional sex; changes in drug use; integration in and isolation from community; overdose prevention; and housing.

### Study Eligibility

Study eligibility required participants to be at least 18 years of age and self-reported to be primarily injecting drugs sold as ‘heroin’ (including heroin adulterated or substituted with fentanyl), either living in/commuting to the research area. The study has been approved by the University of California San Francisco’s Institutional Review Board. We have changed all names for confidentiality.

### Recruitment and Sampling

We used purposive sampling (Barendregt et al., 2005), aiming to recruit as near to equal numbers of men and women as possible since women are under-represented in research on illicit drug use (Tuchman, 2010). For such sampling, the number of interviews needed for data saturation is difficult to predict; however effective saturation has been demonstrated after approximately 12 interviews with a fairly homogeneous sample. For more heterogenous samples, larger numbers may be needed. The research team recruited participants at the SSP and obtained oral consent. Interviews were undertaken upon recruitment privately in or near the SSP and took approximately one hour. Participants received $20 cash for their time. Following the research visit, four recorded phone/video interviews were held with health center staff. These were informational interviews addressing how clients could learn about and access PrEP, any financial requirements, PrEP receptivity and obstacles to uptake. No hypothesis generation was sought from the service provider interviews and data saturation was not a goal. This information provided background to this paper and no payment was made to these interviewees.

### Analysis

The 26 interviews with PWID in WV were professionally transcribed in full and for each we produced an analytic memo of significant observations using inductive and deductive methods (Christopoulos et al., 2015; Strauss & Corbin, 1990). The semi-structured interview guide was used to create an initial memo structure to guide analysis while emergent themes were added as analysis proceeded (see Christopolous et al., 2015). We then re-analyzed the data to reflect these new themes and sought negative cases for comparison (Hamilton, 2020). To allow thematic/content searches across the analysis we assembled the memos in a Word table. We also produced thematic memos addressing findings across the interviews.

While some interviewees emerged as distinctively matching particular modes of social organization, others required more data than was feasible from one interview. Nonetheless, we found trends towards certain modes and indications for further research. For the four staff member interviews, notes were taken of significant informational points from the recorded interviews.

## Findings

### HIV Outbreak and Risk Perception

The sample of 26 people who inject heroin included 18 people who were HIV negative and 8 people diagnosed with HIV in the last year. Those who said they were HIV positive volunteered the information spontaneously without direct questioning. Those who were HIV negative either said so explicitly or made it clear through other answers. Equal numbers were living housed and homeless on the street; among those housed, housing stability ranged from home ownership to shared rental accommodation. Eleven were cisgender female and 15 were cisgender male. Twenty-five described themselves as Caucasian or white, one did not respond.

A striking finding was that, among the 26 interviewees who injected heroin amidst an ongoing HIV outbreak, none had ever taken PrEP, despite it being freely available from the health center where study recruitment took place. By contrast, several of those who had recently tested positive for HIV volunteered that they were taking anti-retroviral treatment.

Only two of the 18 HIV− interviewees did not initially know what PrEP was, some were unclear or had a vague notion of PrEP’s purpose and twelve knew of PrEP’s function as a medication that prevented HIV transmission. Of these, only one person mentioned that she had sought it unsuccessfully from her doctor without disclosing that she injected drugs. Among the eight living with HIV, two had only learnt about PrEP after testing positive in recent months and most expressed surprise that they had acquired HIV. Of the eighteen participants who said they were HIV−, half said they weren’t interested in PrEP because they were not at risk from HIV.

Lack of knowledge of PrEP’s function therefore did not explain the lack of uptake. More puzzling is the low risk perception among the majority of interviewees (15/26). This paper focuses in particular on why people who were injecting drugs amidst a local injection-transmitted outbreak considered themselves currently or previously (prior to infection) at low risk for HIV.

Samantha, a woman in her 20s living homeless and recently diagnosed with HIV, recounted how from early childhood her mother had been ‘pimping me out or selling me to her dope dealers and her johns’ and ever since had been ‘doing dates’. She had injected various opioids for over a decade but expressed shock at her recent positive test result as she had not considered herself at risk from acquiring HIV beforehand: ‘Well like before I found out, uh, that I had it—see, the first test I took came back negative […] and then 2 weeks later it came back positive. […] I never would have thought that I would’ve, you know, gotten it. I don’t know, it was a surprise though, I know that.’ She attributed the transmission to a friend who had given her some of her drug solution by injecting it into her syringe (known as ‘piggybacking’, reportedly a common local practice) but who had not divulged her HIV+ status until later confronted.

Megan, homeless until a few weeks earlier, had been recently diagnosed with HIV and was followed soon after by her partner. Asked whether she had heard about PrEP, she responded, “Yeah. I heard of PrEP – I didn’t think I needed it.” She attributed her perception of low risk to not sharing her syringes or injecting equipment. This was a common response among interviewees. However, on probing, many participants admitted to an exception: sharing with an intimate partner, explaining that sharing syringes and having unprotected sex with their partner did not count as HIV risk behavior. Megan gave a typical answer:

I:So, before you found out, did you think yourself at risk?

P:No because I don't share needles with anybody.

I:Right.

P:Really. I mean, I've shared needles with my boyfriend but I don't share with anybody else.

Caleb, in his 20s, was negative for HIV and explained that he had broken up with his longterm girlfriend a few days earlier. He was living homeless and gave a similar response:

I:[…] Um, but what about with injecting, do you share injecting with anyone?

P:No, absolutely not.

I:Never?

P:No. I mean, I’ve gave people my needles […] but I – I never, I mean, I’ve set up after my old lady, my girlfriend, yeah, I’ve done it after her just because I know what she got, you know what I mean?

I:Yeah, cause she doesn’t have anything?

P:Um, she’s actually – I never had hepatitis until I got with her.

Megan and Caleb’s responses are not only significant in terms of their described syringe sharing behavior but their initial declarations that they did not share with anyone, later modified to acknowledge an exception for intimate partners, was common among couples. During the interview, Megan experienced a sudden realization that the source of her HIV infection was reusing syringes she had gathered to return to the health center in exchange for new ones. Caleb also reflected on his acquisition of hepatitis C within his relationship.

### People Living Homeless

A perceived low risk of acquiring HIV, whether looking back to before it happened or in the present if a person was HIV negative, was most common among people living homeless. People injecting drugs and living on the street tended to follow two social formations:

#### The fatalist ‘street family’

These individuals (*n* = 7) tended to be single and relied opportunistically on fellow members of the ‘street family’, a loose grouping of people living homeless and using drugs, for finding shelter, drugs and other essentials. Brought together by necessity rather than choice, members of the street family frequently attested to a lack of mutual support, frequent stealing from each other and mistrust. Dorie, who was HIV−, answered questions about who she considered ‘family’ and trust:

I:[…] And are there any other people who you consider family who aren’t like –

P:Just my street family.

I:[…] And do you like trust those people or –

P:Not really.

I:No. Okay. Are there any particular people who you do trust on the street or is it – ?

P:No […] you don’t trust no one.

Several people who considered themselves part of the street family expressed a longing for greater solidarity and community but essentially, despite their physical proximity, they constituted isolated, constrained individuals and were prone to fatalism. Fatalism softens the potential for self-blame among people who are marginalized but may also work against PrEP uptake as acquiring HIV may be considered outside a person’s control. Corresponding with a fatalistic explanation of events, members of the street family often blamed conspiracies, both terrestrial and supernatural, for the local HIV outbreak. Sylvie, in her 30s, living homeless and HIV+, explained that the recent outbreak was not due to high risk sexual behavior or syringe sharing but an intentional plan by established members of the community:

[…] And it’s not that we share needles or that we all, you know, have sex with each other or anything like that but somehow someone is sneaking some – […] Honestly, I think that someone was smart enough to figure out how to eventually you know and it may take a good, long while, but how to, get rid of the fucking homeless, junkie problem that has become [the town], you know.

#### Homeless Enclavist Couples

Among those living homeless, six interviewees were in heterosexual couples and none described themselves as part of any other kind of sexual relationship. Of these, four were enclavist. The street family’s pervasive mistrust of each other contrasted with accounts of these intimate partnerships. Trust and exclusivity could be based on norms related to sexual monogamy, reliability to reverse overdoses and/or exclusive sharing of drugs. For instance, Megan explains that she used heroin almost exclusively with her boyfriend because, while he would reverse an overdose, ‘I don’t trust anyone else to make sure that if I were to get far out or something, that they wouldn’t leave’. Danny, in his 40s, injecting heroin for 2–3 years and HIV−, described how he only uses drugs with his wife ‘We look at it as distrust when one goes out and tries to do drugs and the other one doesn’t get any share of it.’

However, despite the presence of trusting relationships in enclavist couples, the lack of housing and enforced socializing from outdoor living as well as the chaotic influence of dependent opioid use and lack of resources prevented their formation of truly separate enclaves. Despite these difficulties, they made some attempts at drawing a boundary around themselves and against the outside (see Fig. 1). Lisa, who shared a tent with her husband Dudley (both HIV−) distinguished herself from other people living homeless. She explained that she avoided those outside the local day center for homeless people that provided showers and meals:

I try to stay away from there, I mean, I just, I don’t know, I’m no better, I know, than anybody else, but I just see some of these homeless people out here and it’s like, they embarrass the shit out of me! And I’m probably not doing – I’m not in any better shape than they are, but I don’t know, I just kinda try to carry it a little different […] just because you’re homeless doesn’t mean we’re helpless, take a bath!

Lisa described her exclusive preference for her husband’s company over those of any other people living homeless:

I:Okay, so who do you normally hang out with during the day?

P:Uh, nobody, my old man. Other than that, I mean, I go and get my personal, you know what I’m saying, and go back home [their tent]. I don’t have a lot of friends cause them are very far, few and in between. People that are out here on the street are all about themselves and getting high and just not my friend. And I ain’t got time for a bunch of fake people, you know, I’m above all that bullshit. So I just – I’m a loner, mm-hmm.

Dudley was asked about the social scene in the homeless encampment where he described their exclusive sharing of his and his wife’s key resource, heroin, and the experience of getting high:

I:And do people hang out, uh, and use together [in the homeless tent encampment]?

P:Yeah […] but, I mean, no one really shares heroin. It’s not that way. I mean, my wife and I do, but other than that, you know, it’s fend for yourself […] and it’s not a social – no, it’s, I mean, I may score something for you or something just to make something, but as far as hanging out and doing it, no.

Observing the way people in couples, such as Megan and Caleb, initially discounted sharing injecting equipment within their relationship as of any significance, we term this an ‘invisible risk group’: those inside the boundary, usually an intimate partner, are discounted as potential vectors of HIV, in spite of evidence to the contrary, while the threat of HIV was largely perceived as lying outside of the boundary of the monogamous relationship.

### Housed Couples and Individuals

Those with stable housing followed two main social formations: (1) the ‘enclavist couples’, like their homeless counterparts, limited their social interactions with others but, in contrast, had stronger boundaries against the outside; (2) the ‘mixers’ were couples and single people who mingled with a very broad group including people who used drugs, friends who did not and were housed or living homeless. The mixers did not have a strong boundary around themselves. We also interviewed three men who lived alone and tried to avoid spending time with the street family but their responses were somewhat contradictory and we lacked sufficient data to confidently classify their social strategies and allegiances. Three others were indeterminate and required further data collection. Perception of HIV-related risk tended to be higher among those housed.

#### Housed Enclavist Couples

Among the couples who were housed, the physical barriers of walls and lockable doors as well as access to private space made enclave-like separation from the outside world more feasible than for their counterparts living on the street. Four interviewees described current enclavist behaviors and ways of ordering their lives and one described earlier enclavist leanings before separation from his partner.

Patricia, a woman in her 30s who had used heroin for about a decade, was sharing an apartment with her husband. As with the couples living homeless, Patricia’s boundary included herself and her husband. Although she mentioned that she sometimes used drugs with others, mostly she was either alone or with her husband and avoided going out on the streets, buying her drugs exclusively from ‘a couple [of dealers] I have a good relationship with’. Trust issues and the risks of drug use were influential on narrowing her social world.

I:Okay. And you said earlier that you use only with your husband?

P:Yes […] I mean, every now and then, you know, we’ll have a friend over at the house and they’ll get high with us but most of the time just him. […] I’m just particular about that cause I don’t want somebody OD-ing in my house or anything like that, you know, and you can’t trust anybody most of the time anymore […] so I just tell him I’ll just use with him.

Patricia, who was hepatitis C positive but HIV negative, reported only sharing syringes with her husband and getting tested for HIV frequently. Although none of the housed participants were using PrEP either, anxiety about acquiring HIV was more evident, both among those in couples and people who were single. Patricia was asked,

I:What are your thoughts about HIV?

P:Um, it’s a scary thing, you know. I mean, I think, you know, no one is safe from it. If you’re an IV drug user it’s definitely something to be worried about and to be cautious about, especially more so if you share needles.

Patricia recounted her effort to police the enclave boundary in a recent incident:

I got onto my husband the other day cause he was gonna use a needle that was someone else’s. I just, we weren’t home – I took off and started walking to the house cause I was pissed off. And he was like, “Well, he said that he’d only used it once and he only has Hep C.” I’m like “You don’t know that for one, for two, he could think that’s all he’s got and he never has gotten tested for HIV or anything else.”

#### Housed Mixers

Mixers (*n* = 4) could be described as ‘weak group’ as they did not have strong boundaries around themselves, socializing with a range of people across varying social contexts. Although there was insufficient data from the interviews to determine whether they were weak or strong grid, they seemed to rely more on individualistic behavioral modification to manage risk than trusting their community or enclave.

Jamie, HIV− and in his 30s, lived with his girlfriend and children and worked in construction. Their active social life included neighbors, a religious group and people using drugs, both housed and living homeless. He explained that he got tested often for HIV, seeing injecting drug use as a high risk. Describing his partner’s sexual risk, he commented with pride, ‘I don’t have one of those “runaround girls”’, yet unlike the invisible risk groups, he did not assume that she was safe to share syringes with and insisted that she too be tested regularly.

I:So, do you ever get tested for HIV or Hepatitis C?

P:On the regular. […] Every three weeks I get an HIV test. Every two months I get a full panel.

I:Oh, right. And your partner – your girlfriend, does she get tested as well?

P:She has to. Same time.

I:Why is that?

P:’Cause I say so.

I:And what – and why do you get tested so often?

P:Um, security. […] I’m an IV user. The biggest thing you can do wrong is be an IV user. That is the most susceptible to anything.

Dave, HIV−, in his 30s and housed, similarly spent time with different friends, both involved in drug use and not and seemed to consider his risk of HIV high. He explained that he was currently avoiding sex entirely due to the local HIV outbreak and also had lost interest due to his heroin use. He commented that since his release from jail, *‘*I’ve not been sexually active cause I’m afraid to friggin’ sleep with any of these women around here […] HIV’s on the rise.’ Later during the interview we discussed PrEP and Dave saw the potential to end his self-imposed celibacy:

I:Have you heard of PrEP?

P:PrEP?

I:Yeah.

P:Yes. They asked me if I’d take PrEP here [at the SSP].

I:Yeah.

P:That’s the way I’ve heard of it.

I:Oh right, and you don’t use PrEP?

P:No. What is PrEP? Is it an AIDS thing?

I:Oh, it stands for Pre-Exposure Prophylaxis. So it’s a pill that you take every day.

P:Okay.

I:And then if you’re exposed to HIV somehow.

P:Yeah.

I:Um, then you don’t –

P:Why haven’t I been told about this? […] I need it. I want it. […] Yeah, cause, here we go, maybe I can get laid.

Like Dave, during the course of the interviews, four of the housed participants expressed interest in getting a prescription for PrEP and a couple who were living homeless also said they were considering it. Those who were housed were more likely to see themselves as at higher risk of HIV transmission than those living homeless and that this risk was something that could be controlled by individual behavior based on, or at least justified by scientific sources or risk assessment of observed behavior.

Abe, a small business owner, lived alone in a rented apartment and was HIV−. He showed characteristics of an individualistic entrepreneur without strong group allegiance or rules. Such individualists in Cultural Theory terms tend to see risk as something to be managed through personal agency, for instance through basing their behavior on knowledge of scientific evidence, rather than on shared community rules. Abe showed his allegiance to science as a basis for decision-making, quoting his knowledge of the evidence for PrEP’s efficacy and decrying the rejection of scientific evidence by climate-change deniers. However, he also viewed his risk of HIV transmission as low based on his own assessment in which he considered sexual behavior between men to be riskier than parenteral transmission:

I:Have you heard of PrEP?

P:I have. I heard it’s something to help […] – it gives you a 50% – 70% chance I think as far as intravenously catching HIV. It prevents – like is that what it does? […] I think the guy here told me 50% to 70%, somewhere in there, like that sounds pretty damn good, I mean, honestly, and he said the side effects are minimal. […] So, I mean, it sounds like a pretty good and he said for you know, sexually transmitted he said it was really high like 90 something percent.

I:Right. Right.

P:So, which I – I don’t worry, I mean, I’m not saying that I’m 100% but I don’t have anal sex really. I’ve done it with, you know, a woman before, but it’s not common and, um, I’m not homosexual. I’m not saying that eliminates me, but it does reduce the risk a whole lot, so […] And plus I don’t sell – I don’t sell, you know, have sex for money or anything like that, so.

Abe’s response may reflect the greater propensity of individualists to take risks when they consider that they could be personally advantageous and controllable. However, during the course of the interview he reconsidered and expressed an interest in taking PrEP, perhaps reflecting receptivity to information from university researchers.

### The Significance of Housing as an Influence on Risk Perception

Even among those who were housed, poverty, intensified by the demands of opioid dependence, affected many of the interviewees. While poverty constrains individuals in many ways, housing or its absence has perhaps the starkest impact on shaping the options available in terms of controlling resources as well as limiting or enabling social relations and the values that grow from and reinforce them. Stable housing allows people to choose with whom and when they associate and share their resources.

The risk of theft of their belongings was a common theme among the interviewees who were living homeless. Mark was HIV negative and living homeless. Though interested in taking PrEP, he explained how homelessness interfered with taking medications regularly:

I:[…] Have you, uh, have you ever heard of PrEP?

P:Yeah […] That’s the shit you take and it kinda helps you, uh, not get it.

I:[…] It prevents HIV. Have you ever used it?

P :No. I’m going to use it when I get my place though, because I’m really bad at – I’m taking my medicines now and I don’t want that to be, um, something that, uh, another thing that I’m not, uh, taking right because, um, I’m on the street, you know what I’m saying? And, you know, more, uh, attentive to my stuff when I’m able to be there at all times […] So I miss my medicines and stuff, so –

The ability of a housed person to control access to personal resources whether through intentional sharing or the prevention of theft may be significant not only in practical terms but in shaping HIV risk perception. Those housed were more likely to perceive HIV as a significant risk and more receptive to the idea of taking PrEP to control their risk. Accordingly, they were less prone to fatalism, with its perceived lack of control, particularly compared with the single members of the street family.

## Limitations

Despite the richness of this data, it was gathered during 1 week of pilot research and due to the small numbers, data saturation was not reached on all points. Future studies could include second interviews, ethnography and larger numbers for each mode of social organization. Potential social desirability bias may have resulted from the institutional setting but interviewers tried to minimize this by asking similar questions in different ways. In common with all qualitative research, further exploration is needed to understand whether similar phenomena exist more broadly.

## Discussion

Most of the participants in the study were either isolates or were forming or attempting to form enclaves and as such would have been described by Douglas as located along the negative diagonal of rejection. While none of the interviewees had ever taken PrEP, their reasons for not doing so varied. Most commonly, they had not or did not currently see themselves as at risk or considered themselves able to control their risk of acquiring HIV, beliefs that had a number of different origins, from the perceived protection of an exclusive social boundary or ‘second skin’ by enclavist couples to the belief in personal control of risk through behavior by housed individualists and mixers. Fatalists, who tended to be living homeless, were less concerned about HIV and as shown in our earlier publication often dismissed it as a significant threat (Mars et al., 2022). The causes of transmission were frequently seen as beyond their control, sometimes arising from supernatural or human conspiracies (Mars et al., 2022). In these ways Cultural Theory was predictive of our findings.

The conspiracy theories described and the forms they took among the street family were also predicted by Douglas and Calvez and illuminate the fatalistic view of HIV prevention held by some. These explanations of causation also served to locate blame outside local people who injected drugs, sometimes redirecting blame towards those on the positive diagonal of hierarchy and individualism. However, the formation of enclaves appeared to be not so much in opposition to the positive diagonal of those perceived as holding power, as predicted by Douglas’ model, but as an intentional separation from the street family, who were perceived as a risk for theft, betrayal and violence.

This divergence from the model also reflects a point of departure between Douglas and some of her collaborators. Michael Thompson, for instance, argues that each mode of organizing is also a way of disrupting and subverting the other modes and that each have distinctive ways of rejecting information that they see as a threat (Thompson, 2008). Accordingly, rejection is not limited to the opposing diagonal but can be directed at any other mode of organization.

The concept of community as a second skin in the prevention of HIV transmission was just as relevant in 2019 West Virginia as in Marcel Calvez’s 1990 Brittany and helped to explain low risk perception and decision-making among enclaves and enclavists. This second layer of protection outside of the body made up the enclave’s boundary and created an ‘invisible risk group’ inside, while risk was viewed as located outside the boundary. While the extent to which possible separation varied, enclavist couples tried to create a ‘safe’ sphere in which there was exclusive sharing of injection equipment, sexual intimacy and drug supplies, with partial success. The second skin gave enclavist couples the impression of safety from acquiring HIV while, on reflection, they conceded the potential for errors and breaches in the boundary. Understanding how social formations and beliefs arise and relations between modes of social organization can therefore help to explain why the use of scientific knowledge alone is insufficient to persuade some of those in high risk situations to take pre-exposure prophylaxis medication.

Our pilot study’s findings did not reach data saturation in all dimensions and, while not definitive due to the low numbers, provide indicative evidence that differences in HIV risk perception may play an important part in decisions around PrEP uptake. These differences are not free floating but arise from the material conditions in which the participants live and the social relations that they enable, limit or negate. Housing access was not considered in the original work by Douglas and Calvez but we found differences between those who were housed and those living homeless both in their capacity to separate from others and in their perceptions of HIV transmission risk. Housing not only allows many more choices and a greater degree of control, it may also reduce the sense of exclusion from established power and its attendant knowledge values.

While street homelessness forces single people out into a community of their peers and reduces the ability of couples to live apart from the ‘street family’, housing allows people to develop a greater sense of control over life decisions thus limiting fatalistic tendencies. Housing also affects the extent to which intentional separation from others, the active choice of allegiances and drawing of boundaries, is feasible. Those who were housed voiced more anxiety about acquiring HIV than those who were living homeless and were more likely to see HIV as a risk they could control through personal agency. Their less alienated position as housed citizens of the town may have made them more receptive to establishment scientific evidence and public health strategies. They showed greater receptivity to the benefits of PrEP, with several expressing an interest in getting a prescription during their interviews. As well as the greater actual and perceived sense of control provided by housing, having a stable living situation has specific practical benefits for safeguarding medications against theft and for maintaining a regular daily dosing regime.

Persuading people who use drugs that they are at risk of acquiring HIV and could benefit from pre-exposure prophylaxis needs to take into account their varying social relations and organization and the values that arise from and reinforce these. While some of the interviewees were receptive to information about PrEP, as has been found in other studies (Zhang et al., 2019), others were not. Scientific data generated by institutional hierarchies and individualists is unlikely alone to persuade or form a basis for decisions by those excluded from or rejecting the establishment from which such evidence emanates. Fatalists are probably the most difficult to engage due to beliefs that their own actions are not decisive in HIV transmission.

Regaining a sense of control in fatalists’ lives is likely to be critical in engaging members of the street family. Access to stable housing, effective treatment for drug dependence and the prospect of legitimate, rewarding employment are key. These are long term goals while the risk of HIV is more immediate. Short term monetary incentives such as those used in contingency management for the treatment of stimulant use disorder (Brown & DeFulio, 2020) could allow PrEP to compete with the immediate priorities of opioid withdrawal, food and shelter. Delivering medication to people or prescribing long-acting injectable PrEP could help overcome some practical barriers (Landovitz et al., 2016). Enclaves also present challenges due to their rejection and mistrust of the establishment as a source of explanations and evidence and their sometimes misplaced trust in the effectiveness of protection from their boundary as was shown during the course of interviews with some of those who had recently tested positive for HIV.

Our findings show conflicting perspectives between the researchers and many of the participants regarding the perceived risk of aquiring HIV with important implications for developing strategies to improve PrEP uptake. Rather than suggesting that an ‘ideal culture’ exists in which we all share the same outlook on science and health, this comparative understanding allows us to see the benefits and drawbacks of each cultural model for its adherents. Cultural Theory offers untapped potential for explaining perplexing phenomena and the ability to make comparisons across different social groupings.

Approaches to engagement need to be varied rather than singular so that they take account of the values and beliefs of people’s contrasting forms of social organization and living conditions. Ethnographic research has great potential as a partner with local health services to identify and tailor the approaches needed for understanding and engaging people within local populations to prevent HIV transmission. Further research using this approach would also be of great benefit in gaining a deeper understanding of risk perception among people who inject drugs and developing strategies for improving their health.

## Declarations

### Originality

This is an original submission that has not been submitted or published elsewhere.
